# PET Imaging with [^18^F]ROStrace Detects Oxidative Stress and Predicts Parkinson’s Disease Progression in Mice

**DOI:** 10.3390/antiox13101226

**Published:** 2024-10-12

**Authors:** Yi Zhu, Neha Kohli, Anthony Young, Malkah Sheldon, Jani Coni, Meera Rajasekaran, Lozen Robinson, Rea Chroneos, Shaipreeah Riley, Joseph W. Guarnieri, Joshua Jose, Nisha Patel, Douglas C. Wallace, Shihong Li, Hsiaoju Lee, Robert H. Mach, Meagan J. McManus

**Affiliations:** 1 Department of Anesthesiology and Critical Care Medicine, Children’s Hospital of Philadelphia, Philadelphia, PA 19104, USA; 2 Department of Radiology, University of Pennsylvania, Philadelphia, PA 19104, USA; 3 Center for Mitochondrial and Epigenomic Medicine, Division of Human Genetics, Children’s Hospital of Philadelphia, Philadelphia, PA 19104, USA

**Keywords:** reactive oxygen species (ROS), neurodegenerative disease, positron emission tomography (PET), oxidative stress, neuroinflammation, mitochondria, MitoPark mouse model

## Abstract

Although the precise molecular mechanisms responsible for neuronal death and motor dysfunction in late-onset Parkinson’s disease (PD) are unknown, evidence suggests that mitochondrial dysfunction and neuroinflammation occur early, leading to a collective increase in reactive oxygen species (ROS) production and oxidative stress. However, the lack of methods for tracking oxidative stress in the living brain has precluded its use as a potential biomarker. The goal of the current study is to address this need through the evaluation of the first superoxide (O_2_^•−^)-sensitive radioactive tracer, [^18^F]ROStrace, in a model of late-onset PD. To achieve this goal, MitoPark mice with a dopaminergic (DA) neuron-specific deletion of transcription factor A mitochondrial (*Tfam*) were imaged with [^18^F]ROStrace from the prodromal phase to the end-stage of PD-like disease. Our data demonstrate [^18^F]ROStrace was sensitive to increased oxidative stress during the early stages of PD-like pathology in MitoPark mice, which persisted throughout the disease course. Similarly to PD patients, MitoPark males had the most severe parkinsonian symptoms and metabolic impairment. [^18^F]ROStrace retention was also highest in MitoPark males, suggesting oxidative stress as a potential mechanism underlying the male sex bias of PD. Furthermore, [^18^F]ROStrace may provide a method to identify patients at risk of Parkinson’s before irreparable neurodegeneration occurs and enhance clinical trial design by identifying patients most likely to benefit from antioxidant therapies.

## 1. Introduction

As the second most common neurodegenerative disease, Parkinson’s disease (PD) has become a pressing public health crisis in modern society. PD is characterized by both motor and non-motor symptoms. The classic motor symptoms, such as tremors, rigidity, and bradykinesia, result from the progressive loss of dopaminergic (DA) neurons in the substantia nigra (SN). While the exact causes are unknown, death of the DA neurons is associated with mitochondrial dysfunction and neuroinflammation. Healthy mitochondria are vital organelles for adenosine triphosphate (ATP) production, providing ~90% of the energy required to fuel the brain. However, dysfunctional mitochondria are a primary source of reactive oxygen species (ROS) [1,2]. Persistent ROS production can cause oxidative stress and damage to subcellular components (DNA, RNA, proteins, etc.) [3]. Furthermore, ROS can directly activate microglia, the resident macrophages of the brain, which in turn produce more ROS via NADPH oxidase (NOX) and nitric oxide synthase (NOS) [3,4]. Thus, oxidative stress represents a critical link between mitochondrial dysfunction and neuroinflammation. Oxidative stress is an early molecular signature that emerges in the preclinical stages of PD before the loss of DA neurons and persists throughout the disease course [5]. Therefore, developing a non-invasive method to track ROS production in the brain could serve as a powerful tool for early detection and monitoring of PD progression in patients.

[^18^F]ROStrace is a positron emission tomography (PET) radiotracer that we specifically developed for imaging oxidative stress in vivo [6,7]. Dihydroethidium (DHE) is the parent compound of [^18^F]ROStrace, which is oxidized by superoxide (O_2_^•−^) and related ROS to ethidium and then becomes trapped in the cell [8]. Due to this trapping mechanism, an increase in ROS within the brain will result in a focal increase in the [^18^F]ROStrace signal [7]. Notably, only the neutral species of [^18^F]ROStrace freely crosses the blood–brain barrier (BBB); once oxidized, [^18^F]ROStrace cannot enter the brain.

The major goal of our current study was to investigate whether [^18^F]ROStrace can detect oxidative stress in conditions that mimic the complexity of PD pathogenesis in most late-onset patients. For this purpose, we employed the MitoPark mouse. The MitoPark phenotype is driven by mitochondrial and immune (mito-immune) stress signaling pathways that represent a major point of convergence between known genetic and environmental risk factors of PD. The MitoPark model recapitulates key prodromal features prior to motor impairment caused by DA neuron loss [9]. MitoPark’s clinical relevance is extended by the recent discovery that variation in mtDNA maintenance genes influences the risk of late-onset PD [5]. MitoPark mice are generated by the selective deletion of the Transcription Factor A Mitochondrial, *Tfam*, in DA neurons. TFAM is a mitochondrial DNA (mtDNA) binding protein that regulates nucleoid architecture and mtDNA stability. Loss of TFAM leads to the release of mtDNA [10] and constitutive activation of STING signaling to IRF3 [11], neuroinflammation [12], progressive nigrostriatal deterioration, and L-DOPA-responsive motor impairment [13]. The crucial role of mitochondria-induced inflammation in MitoPark pathogenesis is found in reports that manganese, which magnifies mito-immune stress signaling via sensitizing the cGAS/STING pathway [14,15,16] and increasing ROS, also accelerates the MitoPark phenotype [17]. On the other hand, drugs that suppress the mitochondrial O_2_^•−^ signal attenuate neuroinflammation and halt the progression of MitoPark PD [12]. Taken together, the results suggest that mitochondrial oxidative stress is a key driver of MitoPark PD pathology via an inflammatory pathway [18].

Therefore, we hypothesized that [^18^F]ROStrace retention would correlate with PD-like symptoms and increased neuroinflammatory burden in the MitoPark mice. To examine this hypothesis, wild-type (WT) and MitoPark mice were imaged with [^18^F]ROStrace microPET from the prodromal phase (2–3 months) to end-stage of disease (6 months). The relationship between [^18^F]ROStrace and PD-like pathology was further explored by metabolic, behavioral and histological analysis conducted in the same animals (Figure S1). Collectively, our data demonstrate [^18^F]ROStrace was sensitive to increased oxidative stress caused by mitochondrial dysfunction and neuroinflammation in MitoPark mice, which occurred early and persisted throughout the disease course.

## 2. Materials and Methods

### 2.1. Animal Models and Experimental Scheme

MitoPark (DAT^+^/Cre: TFAM LoxP/LoxP, C57BL/6 background) mice were kindly provided by Dr. Nils-Goran Larson at the Karolinska Institute, Stockholm, Sweden (now at Max Planck Institute for Biology of Aging, Cologne, Germany). The generation and characterization of MitoPark mice have been described in detail [13]. MitoPark and control mice (WT) were group-housed in a ventilated caging system with ad libitum access to food and water. The environment was temperature (24 °C) controlled and on a 12 h light/dark cycle. All animal care and use in this study was performed under protocols approved by the Institutional Animal Care and Use Committee (IACUC) at Children’s Hospital of Philadelphia Institute. The general experimental scheme is shown in Figure S1. Briefly, animals (WT and MitoPark) from each age group were first subjected to a battery of behavioral studies. Then, an average of 200 µCi of [^18^F]ROStrace was given to each animal via tail vein injection. After a one-hour dynamic PET scan, the animals were recovered overnight. There was no toxicity observed in animals treated with the radiotracer or the parent compound, DHE.

### 2.2. Open Field (OF) Test

OF testing was conducted as previously described with minor changes [19]. Mice were acclimated to procedure room conditions for at least 1 h prior to testing. They were then placed in a clear-walled, square arena (43.2 × 43.2 × 35 cm) for a trial of 15 min. Each trial was video recorded. Movement over time (xy coordinates), time spent in the center zone, latency to approach the center, and total rears were measured. These measures were quantified by Ethovision XT v9.0 software.

### 2.3. Metabolic Analysis

Comprehensive Lab Animal Monitoring System (CLAMS) was applied to monitor changes in animals’ metabolism and circadian rhythmic activity in the home cage environment [20]. The calorimetry system measures the concentrations of O_2_ and CO_2_ at both the inlet and outlet ports of a chamber that channels a specific volume of air. These data allow for the calculation of O_2_ consumption, CO_2_ production, and the respiratory exchange ratio (RER), which in turn yield precise and consistent information regarding overall energy expenditure of the animal. Infrared (IR) beams from the walls of the cage continuously measure the animal’s activity. CO_2_ concentration difference (∆CO_2_), RER, and energy expenditure (KCal/h) from age-matched WT and MitoPark mice were recorded over 4 days with ad libitum access to food and water.

### 2.4. Tapered Balance Beam Test

Balance beam testing was performed as previously described with minor changes [21]. Mice were acclimated to the procedure room and individual home cage for at least 1 h prior to testing. During a three-day experiment, age-matched WT and MitoPark mice underwent a series of five trials each day, which were recorded on video. The first two days served as training days for the mice to cross a beam, during which they were gently nudged to encourage them to do so. On the third day, a grid was added to the beam, and each mouse was required to walk across it without any interference. Performance on the beam was evaluated on the third day by measuring the frequency of paw slips that occurred in the process.

### 2.5. Bead Latency Assay

The bead latency assay was employed to measure distal colonic motility and performed as previously described with minor modifications [22]. In brief, mice were first fasted overnight before starting the assay. After they were anesthetized by isoflurane inhalation (~2%), a Vaseline-lubricated glass bead (2 mm diameter) was inserted into the distal colon (2 cm from anal margin) of each mouse using a lubricated glass rod. Then, mice were placed in clear cages with paper towels at bottom and no food and water accessible. The time required for each mouse to expulse the glass bead was recorded.

### 2.6. Micro-PET Imaging

The radiosynthesis of [^18^F]ROStrace was performed as previously described [7]. Mouse imaging was performed on an Inveon integrated PET/CT scanner (Siemens Medical Solutions, Inc., Malvern, PA, USA). Animals were anesthetized with 2% isoflurane, a tail vein catheter was placed for PET tracer administration, and each animal was placed on the scanner bed. Following injection of 200 µCi of [^18^F]ROStrace, dynamic acquisitions were performed from 0 to 65 min. CT scans for attenuation and image registration were performed after PET imaging for animals imaged on the Inveon PET/CT. PET images were reconstructed using a two-dimensional ordered sets expectation maximization (2D-OSEM) algorithm (Siemens Medical Solutions, Inc., Malvern, PA, USA) as implemented in the manufacturer’s software, including normalization and corrections for radioactive decay, dead time, attenuation, randoms, and scatter. PET images were reconstructed with a matrix size of 159 × 128 × 128 and a voxel size of 0.8 mm (Inveon). Image frame lengths following injection began with a 5-s duration to capture early activity and extended to 5 min at the end of the scan. CT images were reconstructed with a matrix size of 1024 × 1024 × 1016 and a voxel size of 0.09 × 0.09 × 0.09 mm (Inveon) with a manufacturer-supplied reconstruction program.

### 2.7. Micro-PET Image Analysis

PET/CT images were analyzed in PMOD image analysis software version 3.7 (PMOD Technologies Ltd., Zurich, Switzerland). Radiotracer activity concentrations were normalized to injected dose and animal weight to provide semi-quantitative standard uptake values (SUVs) for comparisons. Micro-CT images were manually co-registered and rescaled to the Mirrione mouse MRI-T2 weighted brain template [23], and transformations were applied to micro-PET images to convert them to atlas space, followed by manual adjustment as needed. Whole brain and regional activity concentrations of [^18^F]ROStrace were measured in 10 volumes of interest (VOIs), including cortex, thalamus, cerebellum, hypothalamus, brain stem, periaqueductal gray, striatum, hippocampus, amygdala, and midbrain.

Voxel-wise analyses were performed using Statistical Parametric Mapping 12 (SPM12, Welcome Department of Cognitive Neurology, Institute of Neurology, London, UK; https://www.fil.ion.ucl.ac.uk/spm, accessed on 3 June 2015) and the SPMMouse (http://www.spmmouse.org/, accessed on 6 February 2015) [24] toolbox implemented in MATLAB R2018a (MathWorks Inc., Natick, MA, USA), matching methods in [25] Hsieh et al., 2022. For each comparison group, a voxel-wise two-sample *t*-test was used with an uncorrected *p*-value threshold of <0.005 and a voxel extent of 50, n = 5~13 per group.

### 2.8. Mouse Brain Tissue Preparation

After euthanasia by inhalation of isoflurane, selected WT and MitoPark mice were perfused with cold heparinized PBS (0.2%). Mouse brains were extracted and fixed in 4% paraformaldehyde (PFA) in PBS for 48 h. Then, brains were washed with PBS and moved to cryoprotectant (30% sucrose in PBS) at 4 °C, embedded in the optimal cutting temperature compound (O.C.T) and rapidly frozen with chilled isopentane. Brain tissues were sagittal or coronal sectioned at 12 μm of thickness using a Cryostat (Leica Biosystems, Nussloch, Germany), and slides stored at −80 °C.

### 2.9. Ex Vivo Autoradiography (ARG)

Mouse brains were collected 1 h after [^18^F]ROStrace tail vein injection. Each mouse brain was embedded in the O.C.T tissue block. Then, 30 µm thickness of sagittal tissue section was applied to each tissue block. The tissue slides were exposed to phosphor plates (BAS 2040, GE Healthcare, Chicago, IL, USA) for 24 h. The ARG images were acquired by Typhoon FLA 7000 (West Avenue, Stamford, CT, USA).

### 2.10. Ex Vivo Visualization of Superoxide (O_2_^•−^)-Derived Oxidant Production

DHE is oxidized by O_2_^•−^ and related ROS to ethidium, which can be detected using Ex λ = 532 nm, Em λ = 580 nm [26,27]. To visualize ROS production via microscopy, animals from each age group received 20 mg/kg DHE (DHE stock solution = 1 mg/mL, 100 mg/mL in DMSO, diluted 1:10 with 50/50 mixed Tween 20 and EtOH solution, then further diluted 1:10 with sterile saline) IP, 2 h prior to euthanasia. Mice were perfused with cold PBS, and brains were fixed at 4% PFA in PBS for 24 h in the dark before sectioning. Brains were sliced into 12 μm thick sections, and slides were processed following our immunofluorescence protocol without antigen retrieval. Panoramic fluorescent images of DHE-treated brain tissues were acquired by Keyence BZ-X800 Microscope (Osaka, Japan).

### 2.11. Immunofluorescence (IF) Analysis

After drying slides at room temperature for 10 min, the antigen retrieval procedure (sodium citrate buffer: 10 mM sodium citrate, 0.05% Tween 20, pH 6.0. 30 min at 95 °C) was applied to selected brain tissues. Brain tissues were then incubated with 0.5% Triton X-100 for 15 min. After rinsing with PBS, brain tissues were blocked with 10% bovine serum albumin (BSA) in PBS for 30 min at 37 °C. Selected slides were incubated with primary antibodies (Table S1) overnight at 4 °C. The following day, after rinsing with 1% BSA in PBS, slides were incubated with corresponding secondary antibodies (Table 1) for 1 h at room temperature. All slides were counterstained with Hoechst (1:2000, PBS, Thermo Fisher Scientific, Waltham, MA, USA, H3570) and mounted with VectaShield Antifade Mounting Media (Vector Laboratories, Newark, CA, USA, 101098-042). Images were acquired by using the Keyence BZ-X800 Microscope (Osaka, Japan) and Zeiss 710 Confocal Microscope (Jena, Germany). For astrocyte, microglia and DA neuron quantification, images were uploaded onto Fiji software (version 2.3.0/1.53f), GFAP, Iba and Th positive cells within the ROI (0.1764 mm^2^) were counted by the cell counter plugin, and quantification results were scaled to n/mm^2^. Quantification analysis of lysosomal vesicle storage inside the soma of microglia was performed as previously described [28]. A minimum of 5 individual microglia z-stack images were captured at the SN region from each animal brain section. Maximum intensity z-projections of the image stacks were created by Zen software (version number: 14.0.28.201) (Zeiss, Germany). The area size of each microglial soma and lysosomal vesicle inside soma were manually measured by using Fiji software, and the results are presented as the percentage of microglial soma area occupied by CD68^+^ vesicles.

### 2.12. Circulating Cell-Free Mitochondrial DNA (ccf-mtDNA) Quantification

Ccf-mtDNA quantification was conducted as previously described with minor changes [29]. Blood (100 uL) was collected from each mouse through retro-orbital blood collection, and the plasma collected after sequential centrifugations at 1600× *g* and 16,000× *g* for 10 min each. Protein concentrations from mouse plasma were determined from the resulting supernatant using the Bio-Rad Bradford protein assay (Bio-Rad, Hercules, CA, USA). DNA was isolated from equal amounts of protein using the DNA Clean & Concentrator-5 kit (Zymo Research, Irvine, CA, USA, 11-303). Levels of mitochondrial DNA in the cytosolic enriched fraction were then determined via TaqMan Real-Time PCR assays to detect mitochondrial NADH dehydrogenase subunit 6 (MT-ND6) using the TaqMan probe. 

### 2.13. Western Blot (WB) Analysis

The ventral midbrain (VM) and striatum were micro-dissected and homogenized on ice in 10:1 RIPA lysis and extraction buffer (Thermo Scientific, 89901) with Protease and Phosphatase Inhibitor Cocktail (Sigma-Aldrich, St. Louis, MO, USA, PPC1010). Protein concentrations were determined using BCA Protein Assay (Thermo Scientific) and 16 μg of protein was loaded and separated via SDS-PAGE in NuPAGE 4–12% Bis-Tris Gel with NuPAGE MES Running Buffer (tyrosine hydroxylase (TH) and GAPDH). The proteins were transferred to PVDF membranes using iBlot 2 Transfer Stacks (Invitrogen, Waltham, MA, USA, IB24001) and blocked in 3% BSA in PBS for 30 min at room temperature. Antibodies for TH (ThermoFisher Scientific, PA5-85167) were diluted in 1% BSA (1:1000) in PBS at 4 °C overnight. Antibodies for GAPDH (ThermoFisher Scientific, MA5-15738) were diluted in 3% BSA (1:2000) in PBS for 1 h at room temperature. Subsequently, LI-COR secondary antibodies IRDye 800 CW and IRDye 680 RD were diluted 1:20,000 and 1:10,000, respectively, in 3% BSA in PBS and incubated for 1 h at room temperature. After primary and secondary incubations, TBST (0.1% Tween) washes were performed 3 times (5 min each). Membranes were rinsed in PBS for 5 min prior to imaging in the LI-COR Odyssey, and quantification was performed using Image Studio (LI-COR Biosciences, Lincoln, NE, USA).

### 2.14. Statistical Analysis

All the statistical analyses were performed in GraphPad Prism software, version 7.02 (GraphPad Inc., San Diego, CA, USA), and the results were presented as mean ± standard error of the mean (SEM). The statistical significance between different animal groups was determined with either one-way or two-way ANOVA following Sidak’s or Tukey post hoc *t*-test. *p*-value < 0.05 was considered as statistically significant.

## 3. Results

### 3.1. Increased [^18^F]ROStrace Retention in MitoPark Mice Occurred Early and Persisted to End-Stage Disease

**Figure 1:**
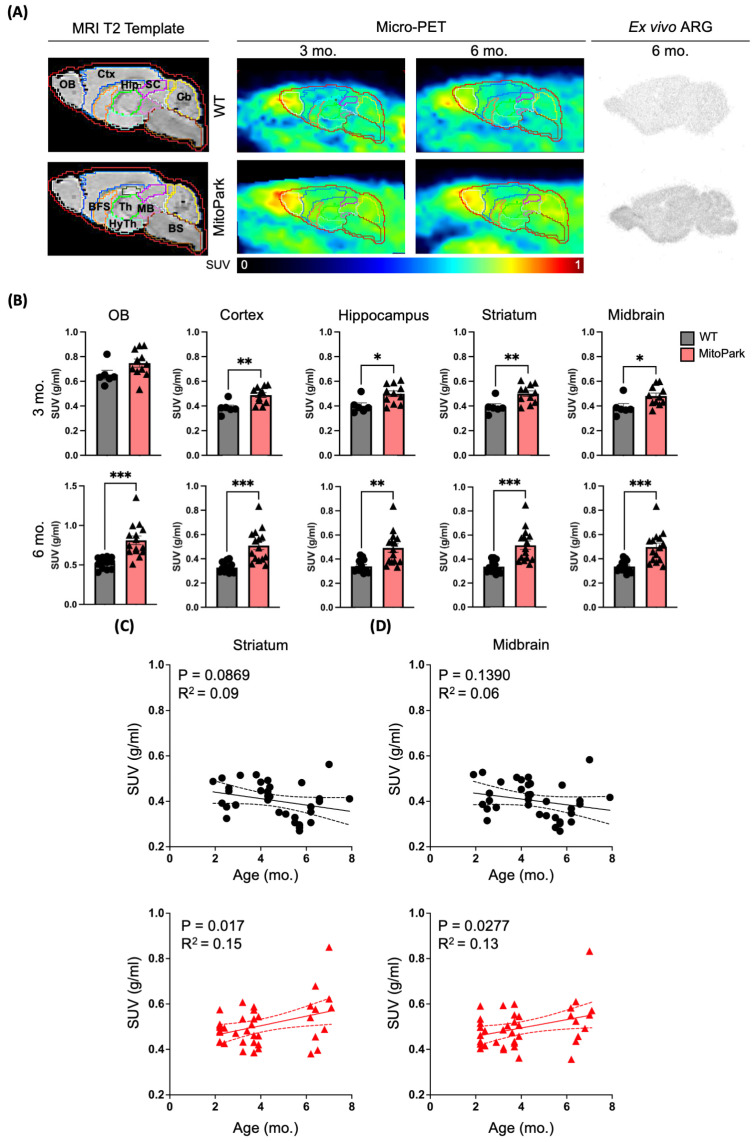
Increased [^18^F]ROStrace retention in MitoPark mice occurred early and persisted to end-stage of disease. (**A**, left panel) Brain subregions derived from Mirrione mouse brain atlas highlighted as follows: olfactory bulb (OB; white), cortex (Ctx; light blue), basal forebrain septum (BFS; orange), hippocampus (Hip; dark green), thalamus (Th; light green), hypothalamus (HyTh; teal), superior colliculi (SC; magenta), midbrain (Mb: pink), cerebellum (Cb, yellow), and brain stem (BS; brown). (**A**, middle panel) Sagittal view of [^18^F]ROStrace PET images showing increased [^18^F]ROStrace retention in the brain of MitoPark mice at 3 and 6 months of age compared to WT mice. (**A**, right panel) Ex vivo ARG validation of [^18^F]ROStrace PET in 6-month-old MitoPark mice. (**B**) Quantification of [^18^F]ROStrace standardized uptake value (SUV) in brain subregions highlighted in (**A**). Values represent mean ± SEM, n = 6–15 per group. *p* values of MitoPark vs. WT determined by unpaired *t*-test (* *p* < 0.05; ** *p* < 0.01; *** *p* < 0.001). (**C**,**D**) Linear regression analysis of WT (black dots, n = 37) and MitoPark (red triangles, n = 39) age vs. Striatum (**C**) and Midbrain (**D**) [^18^F]ROStrace SUV.

We first sought to determine whether O_2_^•−^ is a key trackable signal of neuroinflammation in the MitoPark mouse model of PD. In this model, mitochondrial dysfunction is induced in dopaminergic (DA) neurons by the selective deletion of *Tfam*, which results in progressive degradation of the nigrostriatal DA axis and parkinsonian symptoms that become fatal by 7 months of age [13]. To quantify oxidative stress in vivo, we compared [^18^F]ROStrace retention in the whole brain of age-matched WT and MitoPark mice using micro-PET/CT (Inveon, Siemens Medical Solutions, Inc., Malvern, PA, USA). MitoPark mice exhibited increased whole-brain [^18^F]ROStrace retention as early as 2 months of age (n = 11, *p* = 0.0167), with this increase persisting from 3 months to 6 months of age (n = 15, *p* = 0.0013) (Figure 1A, middle panel). To validate these PET findings, we performed ex vivo autoradiography (ARG) using [^18^F]ROStrace, which confirmed elevated signals in the whole brain of MitoPark mice compared to WT (Figure 1A, left panel). Next, we applied the Mirrione mouse brain atlas (Figure 1A, right panel) to our PET data to assess [^18^F]ROStrace retention in specific brain subregions, focusing on regions either housing DA neurons (olfactory bulb, midbrain) or influenced by the nigrostriatal DA system (cortex, hippocampus, striatum). Our results show that [^18^F]ROStrace retention was increased in the cortex, striatum and midbrain of 3-month-old MitoPark mice (Figure 1B, 3 months), compared to WT. [^18^F]ROStrace signal was further elevated and widespread to the brain regions most associated with the dopaminergic pathway in the 6-month-old MitoPark mice (Figure 1B). To further explore the relationship between [^18^F]ROStrace retention and age, we performed linear regression analyses for the whole brain, striatum (Figure 1C), and midbrain (Figure 1D) SUVs in aging MitoPark mice. These analyses revealed a positive correlation between [^18^F]ROStrace SUV and age in whole brain (*p* = 0.0067, R^2^ = 0.2) as well as nigrostriatal brain regions of MitoPark mice (Figure 1C,D), but not in WT controls (WT whole brain, *p* = 0.0603, R^2^ = 0.1).

### 3.2. [^18^F]ROStrace Retention Correlates with the Gradual Deterioration of Motor and Metabolic Functions in MitoPark Mice

**Figure 2:**
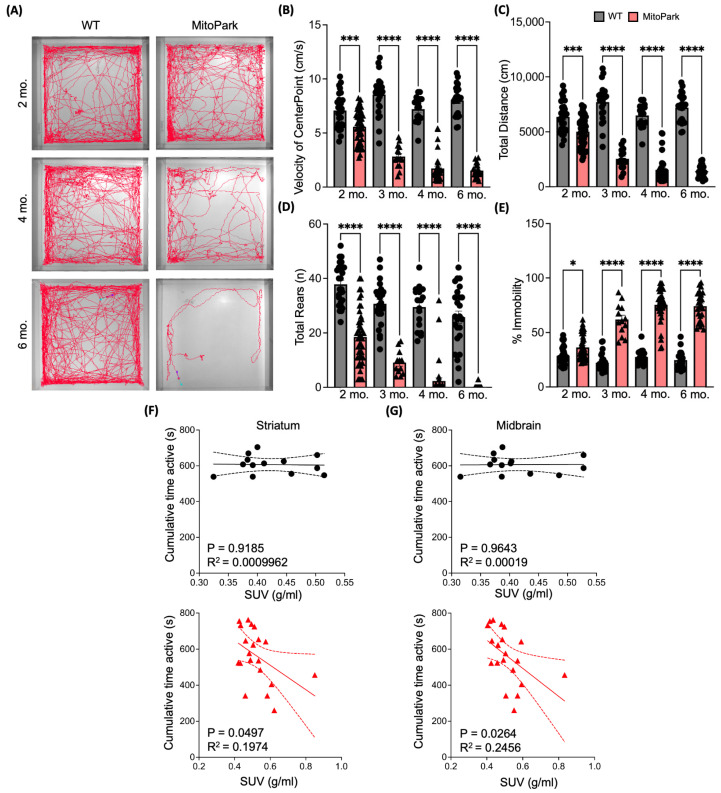
Progressive decline of motor function in MitoPark mice. Representative images (**A**) showing the progressive reduction in distance traveled by MitoPark (right panel) compared to WT (left panel) within 15 min at 2–6 months of age. Quantitative analysis revealed a progressive decrease in motor function determined by reduced velocity (**B**), total distance traveled (**C**), number of rears (**D**), and increased time immobile (**E**) during the 15 min test. Values represent mean ± SEM, n = 15–47 per group. *p* values of MitoPark vs. WT determined by two-way ANOVA with mixed effects model followed by Tukey’s post-test (* *p* < 0.05; *** *p* < 0.001; **** *p* < 0.0001). (**F**,**G**) Linear regression analysis of WT (black dots, n = 13) and MitoPark (red triangles, n = 23) of cumulative time in activity vs. Striatum (**F**) and Midbrain (**G**) [^18^F]ROStrace SUV.

PD is clinically characterized by the progressive deterioration of the patient’s motor system, resulting in symptoms such as bradykinesia, tremor, or stiffness [30]. Therefore, to investigate the relationship between [^18^F]ROStrace retention and PD-associated motor deficits in the MitoPark model, a series of neurobehavioral assessments was conducted. To evaluate gross motor function, we first examined spontaneous locomotor activity in the open field test. Progressively reduced movement was observed in the aging MitoPark mice, as demonstrated by the representative activity tracings in Figure 2A. Motor impairment in the MitoPark mice was further characterized by measuring the velocity (Figure 2B), distance traveled (Figure 2C), rearing (Figure 2D) and percentage of immobile time (Figure 2E) in the open field. Our data revealed a consistent deterioration of motor function in MitoPark mice. To explore the relationship between oxidative stress and PD-related motor impairment, cumulative time spent active was plotted against [^18^F]ROStrace SUV in nigrostriatal regions. A negative correlation was observed between striatum and midbrain [^18^F]ROStrace SUV vs. activity in MitoPark mice (Figure 2F,G), suggesting a link between oxidative stress and motor impairment.

**Figure 3:**
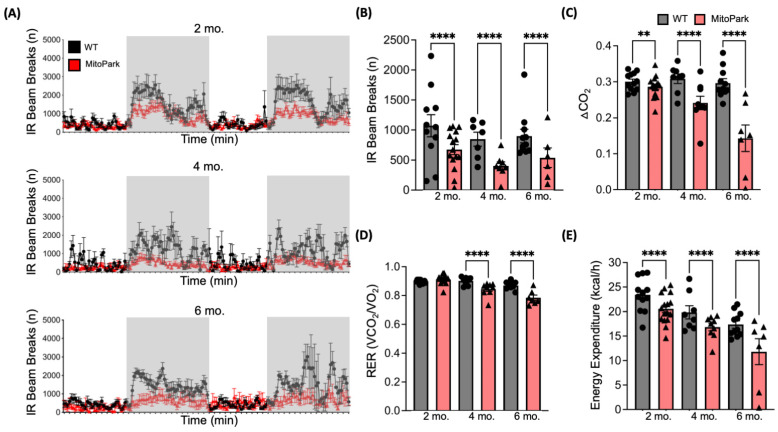
Gradual deterioration of metabolic function in MitoPark mice. Progressive decrease in MitoPark activity calculated as mean number of infrared (IR) beam breaks per time point within the CLAMS home cage during the dark cycle (**A**, shaded regions). Quantification analysis revealed decreased average activity in the dark cycle (**B**), respiration (delta CO_2_; **C**), respiratory exchange ratio (RER, VCO_2_/VO_2_; **D**) and energy expenditure (Kcal/h; **E**) in MitoPark mice over time. Bar graphs represent the mean ± SEM, n = 7–26 per group, *p* values of MitoPark vs. WT determined by two-way ANOVA with mixed effects model followed by Tukey’s post-test (** *p* < 0.01; **** *p* < 0.0001).

Next, movement and metabolism of MitoPark mice were continuously monitored in the home cage environment via the Comprehensive Lab Animal Monitoring System (CLAMS; Columbus Instruments, Columbus, OH, USA). The results again highlighted the progressive nature of the MitoPark phenotype, with significant deterioration of activity (Figure 3A,B), whole body respiration (ΔCO_2_; Figure 3C) and the respiratory exchange ratio (RER; Figure 3D) from 2 to 6 months of age. Similarly, whole-body energy expenditure (kcal/h) in the MitoPark mice was also decreased (Figure 3E) over time. Together, these findings indicate that early detection of oxidative stress via [^18^F]ROStrace can predict the progressive deterioration of motor function in the MitoPark model.

### 3.3. [^18^F]ROStrace Identifies the Male Sex Bias in MitoPark Mice

**Figure 4:**
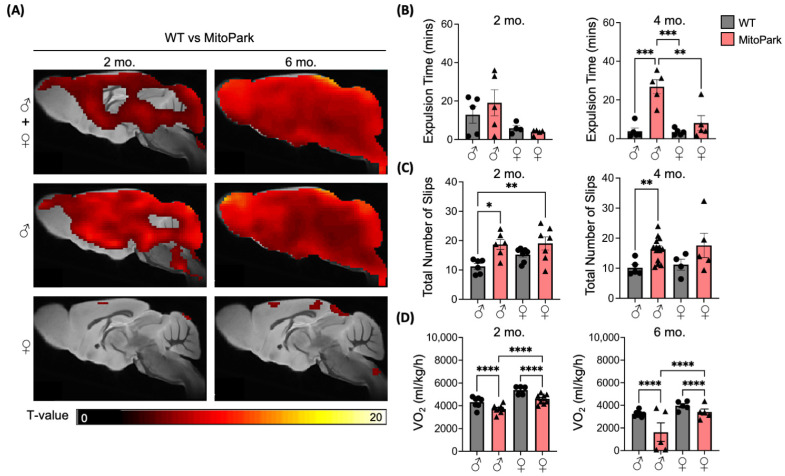
[^18^F]ROStrace detects male sex bias in MitoPark mice. (**A**) Voxel-wise analyses by statistical parametric mapping (SPM; *p* value and threshold) showing the greatest differences in [^18^F]ROStrace retention in male WT vs. MitoPark brains (**A**, middle panel), with MitoPark females more closely resembling WT (**A**, lower panel). (**B**) Progressive impairment of gut motility, measured by latency to bead expulsion, in MitoPark males. (**C**) Impaired motor coordination in MitoPark males from 2–4 months of age, measured by foot slips on a tapered beam. (**D**) Decreased O_2_ consumption by male MitoPark mice vs. WT males and MitoPark females in the home cage environment. Values represent mean ± SEM, n = 5~14 per group, *p* values of MitoPark vs. WT determined by two-way ANOVA followed by Tukey’s post-test. * *p* < 0.05, ** *p* < 0.01, *** *p* < 0.001, **** *p* < 0.0001.

Growing clinical evidence indicates that males are more susceptible to developing PD than females [31,32], which may be partially explained by higher oxidative stress and lower mitochondrial function in males [33]. Consistent with this, multiple PD animal models also reveal that males develop parkinsonian symptoms (rigidity, bradykinesia, and postural instability) sooner than females [34,35]. Therefore, we hypothesized that MitoPark mice may also demonstrate a male sex bias that is detectable by [^18^F]ROStrace imaging. To test this hypothesis, statistical parametric mapping (SPM) was used to distinguish brain regions with elevated [18F]ROStrace signal in MitoPark relative to WT in both sexes (Figure 4A). The SPM analysis revealed that regional differences between the two genotypes were largely driven by the male sex (Figure 4A). Importantly, the brain regions showing increased [^18^F]ROStrace retention in males were key dopaminergic areas, such as the olfactory bulb (OB), striatum and midbrain. Since MitoPark males showed significant differences from WT at a young age (2 months), we sought to determine if male MitoPark mice also show greater changes in behavior related to prodromal PD. Gastrointestinal motility and fine motor coordination were previously shown to be impaired in the “prodromal” stage of rodent PD models [22,36]. Thus, we employed the bead expulsion and tapered beam assays to investigate sex differences in gastrointestinal motility and fine motor coordination, respectively. Young MitoPark males exhibited reduced gut motility (Figure 4B) and impaired fine motor coordination (Figure 4C) compared to WT, whereas female MitoPark were indistinguishable from WT (Bead: 2 months, *p* = 0.9912; 4 months, *p* = 0.6644; Beam: 2 months, *p* = 0.1348; 4 months, *p* = 0.2279). Both male and female aged MitoPark mice were unable to complete these behavioral tasks due to the advanced PD-like phenotype at 6 months of age. However, when the intrinsic stress associated with these tests was eliminated and the mice were observed at baseline in their home cage environment, male MitoPark mice had the lowest whole-body oxygen consumption (VO_2_) at both the early (2 months) and end (6 months) stages of disease (Figure 4D), indicating more severe metabolic symptoms throughout the disease course. Collectively, these results show that [18F]ROStrace effectively identifies the male sex bias in the PD-like phenotype of MitoPark mice.

### 3.4. Oxidative Stress and Neuroinflammation Precede the Loss of Dopaminergic (DA) Neurons in the SN Region of MitoPark Mice

**Figure 5:**
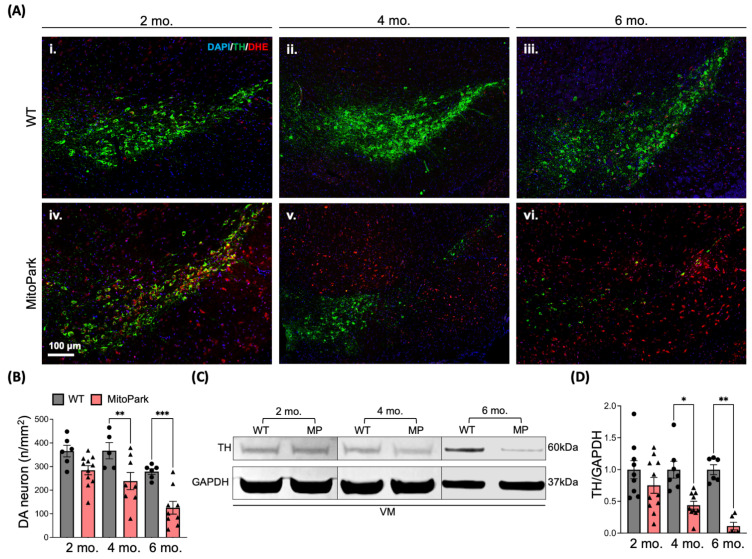
Increased DHE signal in the substantia nigra (SN) accompanied by progressive loss of DA neurons in MitoPark mice. (**A**(**i**–**vi**)) Representative coronal images of the SN from WT (**A**(**i**–**iii**)) and MitoPark (**A**(**iv**–**vi**)) mice at 2–6 months of age showing increased DHE signal and decreased tyrosine hydroxylase (TH)-positive neurons in the MitoPark SN. Scale bar = 100 µm. (**B**) Quantification of TH-positive neurons in the SN of age-matched WT and MitoPark mouse brain tissues. Values represent mean ± SEM, n = 5–11 per group, *p* values of MitoPark vs. WT determined by two-way ANOVA followed by Tukey’s post-test. ** *p* < 0.01, *** *p* < 0.001. Western blots confirmed decreased TH in the SN (**C**,**D**) of MitoPark mice at 4–6 months of age, n = 6–11 per group, values represent mean ± SEM. *p* values determined by two-way ANOVA with mixed effects model followed by Tukey’s post-test. * *p* < 0.05; ** *p* < 0.01.

Previous studies have shown a loss of DA neurons in the SN and increased oxidative damage in MitoPark mice at 6–7 months of age [12]. To further validate our in vivo PET data showing increased early and persistent oxidative stress in the MitoPark brain, we treated WT and MitoPark animals with dihydroethidium (DHE; (20 mg/kg), the parent compound of [^18^F]ROStrace, at 2–6 months of age. When DHE is oxidized to ethidium by O_2_^•−^ or related ROS, it becomes fluorescent (λexcitation/λemission = 532 nm/580 nm) and locked in the cell, thereby indicating local ROS [37,38,39,40]. The fluorescent signal from oxidized DHE and TH+ neurons within the SN region was acquired from age-matched WT and MitoPark brain sections. MitoPark mice exhibited increased oxidized DHE at all time points (Figure 5A), mirroring the PET results. However, loss of DA neurons in the SN region did not begin until 4 months of age (Figure 5A,B), which was confirmed by Western blot analysis of TH levels in the ventral midbrain (VM) (Figure 5C,D). To identify the potential cellular sources of ROS, we examined colocalization of oxidized DHE with cell-type-specific markers for DA neurons (TH), astrocytes (GFAP), and microglia (IBA-1) in the SN region (Figure S2). Our results revealed that oxidized DHE was infrequently detected in astrocytes (Figure S2A,D) in both WT and MitoPark. However, it was localized to the cytoplasm of microglia (Figure S2B,E) and nuclei of dysfunctional DA neurons (Figure S2C,F). These findings suggest that astrocytes play a minimal role in the early [^18^F]ROStrace signal in the SN, with ROS primarily generated by microglia and dysfunctional DA neurons.

**Figure 6:**
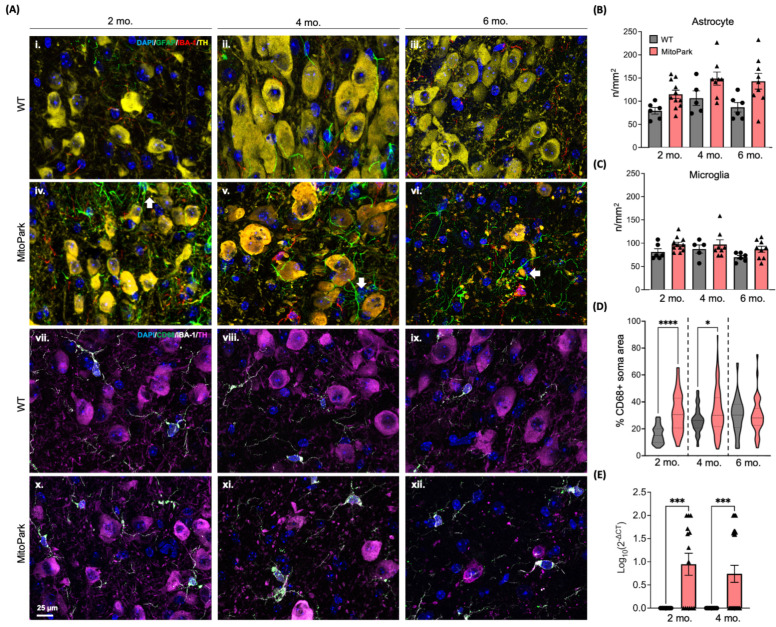
Neuroinflammation preceded loss of DA neurons in the SN region of MitoPark mice. (**A**(**i**–**vi**)) Representative images from the WT and MitoPark SN showing infiltration of astrocytes (green) and microglia (red) surrounding DA neurons (yellow) in the SN region of MitoPark (white arrow). (**A**(**vii**–**xii**)) Representative images showing microglia (white) co-stained with CD68 (green) are increased in the SN of MitoPark at 2–4 months of age. Scale bar = 25 µm. (**B**,**C**) Quantification of astrocytes and microglia in the SN region showing an increased trend in MitoPark mice from 2–6 months of age compared to age-matched control (n = 6–11 per group, values represent mean ± SEM, *p* values determined by two-way ANOVA with mixed effects model followed by Tukey’s post-test). (**D**) Quantification of the % of CD68^+^ microglial soma demonstrating enlarged lysosomal vesicles in MitoPark mice from 2–4 months of age compared to age-matched control (n = 6–11 per group, values represent mean ± SEM, *p* values of MitoPark vs. WT determined by two-way ANOVA with mixed effects model followed by Tukey’s post-test, * *p* < 0.05; **** *p* < 0.0001). (**E**) Increased circulating cell-free mitochondrial DNA (ccf-mtDNA) expression level detected by real-time PCR from plasma of WT and MitoPark mice at 2–4 months of age. n = 14–24 per group, values represent mean ± SEM, *p* values of MitoPark vs. WT determined by one-way ANOVA followed by Tukey’s post-test. (*** *p* < 0.001).

Chronic neuroinflammation is a crucial factor in the progression of PD in both human patients and animal models [41], including MitoPark [12]. Neuroinflammation is intimately tied to oxidative stress. Thus, to better understand potential mechanisms contributing to high [^18^F]ROStrace retention in the MitoPark mice, we examined neuroinflammatory markers in the SN region by IF. Our analysis revealed early infiltration of microglia and astrocytes in the MitoPark SN region between 2 months and 4 months of age, prior to the loss of DA neurons (Figure 6A(i,ii,iv,v)). By 6 months of age, only a sparse population of DA neurons remained in the SN, with astrocytic branches continuing to envelop the surviving neurons (Figure 6A(vi), white arrow). The presence of astrocytes encasing *Tfam*-deficient DA neurons across all stages of degeneration indicates their potential role in supporting these neurons throughout disease progression. Although further quantitative analysis of astrocytic (Figure 6B) and microglial (Figure 6C) densities did not demonstrate statistical differences between age-matched WT and MitoPark in the SN region, a general trend of an increased number of both cells was observed in MitoPark across all age groups.

Recently, single-cell RNA sequencing analysis of microglial phenotypic heterogeneity during the progression of neurodegeneration showed that microglia proliferate in neurodegenerative disease conditions, especially in the early stages, but their morphology and functional changes (e.g., expression of inflammatory markers) are more indicative of disease progression than their numbers [42]. Furthermore, researchers also indicated that functional microglial changes, such as increased phagocytosis and activation, correlate more closely with disease severity than sheer cell number [43]. Therefore, we applied CD68, an endosomal-lysosomal reporter of phagocytosis [44,45], to the brain tissues from age-matched mice. Our results demonstrated increased size of CD68^+^ lysosomal vesicles in the microglia of the MitoPark SN at 2–4 months of age compared to WT (Figure 6A(vii,viii,x,xi)), which appeared to normalize by 6 months of age (Figure 6A(ix,xii)). The increased percentage of microglial soma occupied by CD68^+^ lysosomal vesicles in the 2- and 4-month-old MitoPark mice suggests a heightened phagocytic activity early in the disease process (Figure 6D).

Stressed mitochondria, such as those observed in *Tfam*-deficient DA neurons (Figure S3), can lead to the release of mtDNA, which has pro-inflammatory properties [11]. Circulating cell-free mtDNA (ccf-mtDNA) has been linked to DA neuronal death and neuroinflammation in PD models [15]. Furthermore, we have recently found that the release of pro-inflammatory ccf-mtDNA is regulated by ROS during viral infection [29]. To determine whether a similar mechanism may be involved in MitoPark pathology, we measured and compared ccf-mtDNA levels in plasma from MitoPark and control mice by using real-time PCR. Our results showed that ccf-mtDNA levels were highest in young MitoPark mice (Figure 6E) but became undetectable by 6 months of age, at which point degeneration of DA neurons in the MitoPark SN was nearly complete (Figure 5C,D). Collectively, these results suggest that activated microglia may contribute to increased oxidative stress detected by DHE and [^18^F]ROStrace PET/CT, and these early changes coincide with systemic markers of pro-inflammatory mitochondrial stress.

## 4. Discussion

Evidence suggests a crucial, early role of oxidative stress in the pathogenesis of prevalent neurodegenerative diseases (ND), including Alzheimer’s disease (AD), Parkinson’s disease (PD), and amyotrophic lateral sclerosis (ALS) [46]. Mitochondrial dysfunction appears central to this process, as it can trigger the release of reactive oxygen species (ROS) and other mitochondrial stress signals that activate the innate immune response [47]. Oxidative stress emerges as both a cause and consequence of neuroinflammation, further contributing to the abnormal processing and accumulation of proteins such as amyloid-β (Aβ), α-synuclein, and tau [47,48,49,50,51,52]. As a result, oxidative stress acts as a pivotal player, intertwining mitochondrial dysfunction, neuroinflammation, and proteotoxicity in the complex landscape of neurodegenerative diseases. Oxidative stress can be silently cultivated in the brain of ND patients’ years before the appearance of diagnostic symptoms. This extensive prodromal phase may afford a valuable opportunity for early disease intervention, underscoring the urgent need for noninvasive methods to detect oxidative stress in the brain.

Positron emission tomography (PET) imaging holds great promise for addressing this issue. Several PET probes have been developed to monitor levels of ROS in vivo, and most of these involve a trapping mechanism of an oxidized metallic or ethidium-based radiotracer in tissues with high ROS [53]. Metallic radionuclides have the advantage of simpler synthesis and higher radiochemical yields. Of these, ^68^Ga-Galuminox has shown promise in vitro and in a mouse model of LPS-induced acute lung injury, but brain uptake has not been described [54]. Copper radionuclides have also been described as potential probes for oxidative stress. One example, [^64^Cu]Cu-ATSM, becomes trapped in cells exhibiting high NADH levels [54], which indicates reductive stress. While [^64^Cu]Cu-ATSM may prove valuable for detecting mitochondrial dysfunction [54,55], it does not measure ROS and can accumulate in cells that primarily depend on glycolysis independent of ROS levels. Conversely, Cu chelators such as [^64^Cu][Cu^I^(BCS)_2_]^3−^ do directly detect ROS, but do not cross intact cell membranes and are less stable in blood, thus requiring further optimization for in vivo imaging [56]. The second major class of ROS-sensitive PET probes was developed by our group and is based on the fluorescent probe dihydroethidium (DHE). DHE is an uncharged lipophilic compound that easily passes through cell membranes and the blood–brain barrier (BBB) but becomes trapped in the cell when oxidized by superoxide (O_2_^•−^) and related ROS. Studies in a doxorubicin model of cardiotoxicity involving O_2_^•−^ demonstrated feasibility of the first DHE-based radiotracer for imaging ROS levels in tissues under oxidative stress in vivo with PET. Unfortunately, this radiotracer does not cross the BBB and cannot be used in central nervous system (CNS) imaging studies [6]. The second-generation compound involved the replacement of the “click” moiety for radiolabeling with a 2-fluoroethoxy group to form [^18^F]ROStrace, with a half-life of 109.7 min [7]. Initial in vivo imaging studies of [^18^F]ROStrace were conducted in an LPS-treated mouse model of neuroinflammation. This study demonstrated that [^18^F]ROStrace has a high initial brain uptake (0–3 min), followed by a rapid washout of the reduced form and stabilization of the oxidized form after 20 min. The average whole brain [^18^F]ROStrace SUV during this plateau phase (20–60 min post-injection) was higher 24 h post-LPS and correlated with the sickness score [7]. We have recently validated the utility of [^18^F]ROStrace in multiple models of proteinopathy, including the APP/PS1 AD model [25] and the A53T model of synucleinopathy [57]. [^18^F]ROStrace retention occurred in the initial phase of each proteinopathy, spatially and temporally correlated with amyloid and alpha-synuclein accumulation, and detected increased sensitivity of A53T mice to immune challenge [25,57]. These studies highlight the potential utility of [^18^F]ROStrace for early detection of oxidative stress in AD and synucleinopathies.

Herein, we investigated whether [^18^F]ROStrace could detect oxidative stress in conditions mimicking PD pathogenesis. Overproduction of ROS by stressed mitochondria is implicated in the erosion of the dopaminergic axis and the acceleration of PD pathogenesis [41,58,59]. A phenotyping pipeline was employed to evaluate the relationship between [^18^F]ROStrace and common clinical disease outcomes in MitoPark mice, an established model of late-onset PD induced by mitochondrial dysfunction [13]. We discovered an early increase in [^18^F]ROStrace retention in MitoPark mice that persisted throughout the course of disease. Behavioral studies indicated early GI dysfunction and progressive deterioration of locomotor activity and metabolic function, coincident with elevated neuroinflammation and oxidative damage that was detectable by [^18^F]ROStrace. One limitation of the MitoPark model is the lack of alpha-synuclein pathology, a key feature of PD. However, as aforementioned, we recently found that [^18^F]ROStrace correlates with alpha-synuclein aggregation in a model of synucleinopathy [57], which suggests [^18^F]ROStrace may also be useful for detecting alpha-synuclein-associated neuroinflammation in PD.

Our findings also revealed a noticeable sex bias, with male MitoPark mice exhibiting more severe oxidative stress and PD-like symptoms compared to females. Since the animals used in this study were young (≤6 months), the protective effects of estrogen on mitochondrial biogenesis [60,61], antioxidant defenses [62], and microglial activation [63,64] may have reduced oxidative stress and PD-like symptoms in premenopausal females. The sex-specific difference in [^18^F]ROStrace retention underscores the higher susceptibility of males to PD, in line with clinical observations. PD prevalence is twice as high in males and is often associated with earlier onset and more severe symptoms. The PD male sex bias has recently been linked to advanced brain aging by structural changes in T1-weighted MRI [65]. Experimental models of PD suggest these structural changes may be related to greater mitochondrial dysfunction [66], neuroinflammation [67], and cell death [68] in male subjects. Thus, our results suggest [^18^F]ROStrace PET may be a useful tool to explore these functional changes clinically.

Mitochondrial dysfunction and inflammation are inextricably linked by ROS [69,70,71,72]. Stressed mitochondria release pro-inflammatory damage-associated molecular patterns (DAMPs), such as ROS and mtDNA [73]. In MitoPark animals, deletion of *Tfam* causes mtDNA instability, which may lead not only to the release of mtDNA [10,11], but also to an increase in ROS production from a faulty respiratory chain [74]. Once oxidized, cell-free mtDNA activates the inflammasome [75], resulting in the production of proinflammatory cytokines that promote gliosis, oxidative stress, and neuronal damage [76]. We found increased circulating cell-free mtDNA (Figure 6E) and increased ROS in the SN region of MitoPark mice prior to loss of DA neurons (Figure 5A,B). The increase in ROS detected by DHE was accompanied by activated microglia in the SN region of MitoPark mice, which were absent in the age-matched WT. Although there was no significant difference observed in the number of astrocytes and microglia between the MitoPark and WT SN, astrocytes appeared to play a role in supporting *Tfam^−/−^* DA neurons. Moreover, microglia in the MitoPark SN exhibited greater phagocytic activity, as evidenced by the increased size of CD68^+^ lysosomal vesicles (Figure 6D). The temporal increase in [^18^F]ROStrace retention in MitoPark mice correlated with ex vivo measures of ROS and neuroinflammation in the SN region of MitoPark mice, demonstrating that [^18^F]ROStrace tracks PD-related oxidative stress in vivo.

The early and persistent increase in oxidative stress identified by [^18^F]ROStrace in MitoPark mice suggests that sustained oxidative stress might be a key driver of PD progression. This finding aligns with its established connection between mitochondrial dysfunction and neuroinflammation, highlighting the potential for targeting oxidative stress to alter disease outcomes. Future studies are required to determine whether [^18^F]ROStrace can be used to evaluate the efficacy of mitochondria-targeted antioxidants [50] and other mito-immune therapies.

## 5. Conclusions

In summary, our study demonstrated that mitochondrial dysfunction in dopaminergic (DA) neurons leads to a progressive decline in both metabolic and motor function in MitoPark mice. The PET probe [^18^F]ROStrace proved an effective tool for tracking the abnormal production of ROS during the progression of MitoPark pathology. Notably, increased oxidative stress, as detected by [^18^F]ROStrace, was observed early in the disease course and correlated with disease severity, revealing a male sex bias in the MitoPark model. Furthermore, [^18^F]ROStrace retention was high in young MitoPark mice, prior to the loss of dopaminergic neurons, suggesting the potential utility of [^18^F]ROStrace for the early detection of Parkinson’s disease in at-risk patients.

## Figures and Tables

**Table 1:** List of primers used in the study.

Gene Product	Forward Primer	Reverse Primer	Assay No.
NADH dehydrogenase subunit 6 (MT-ND6)	GCTACTACAACCCTTCGCTGCCA	GGGCTCTTTGGTGAAGAGTTTTATTGC	Mm04225325_g1

## Data Availability

Data is contained within the article or Supplementary Materials.

## Supplementary Materials

The following supporting information can be downloaded at: https://www.mdpi.com/article/10.3390/antiox13101226/s1, Figure S1: Schematic illustration of experimental scheme; Figure S2: Colocalization of oxidized DHE in the MitoPark microglia and DA neurons. Figure S3: Abnormal mitochondria in TH+ neurons of young MitoPark mice; Video S1: 4-month-old WT, tapered beam. Video S2: 4-month-old MitoPark, tapered beam. Table S1: Information for primary antibodies.

## References

[1] Murphy M.P. (2008). How Mitochondria Produce Reactive Oxygen Species. Biochem. J..

[2] Starkov A.A. (2008). The Role of Mitochondria in Reactive Oxygen Species Metabolism and Signaling. Ann. N. Y. Acad. Sci..

[3] Schieber M., Chandel N.S. (2014). ROS Function in Redox Signaling and Oxidative Stress. Curr. Biol..

[4] Lijia Z., Zhao S., Wang X., Wu C., Yang J. (2012). A Self-Propelling Cycle Mediated by Reactive Oxide Species and Nitric Oxide Exists in LPS-Activated Microglia. Neurochem. Int..

[5] Ferrer I., Martinez A., Blanco R., Dalfó E., Carmona M. (2011). Neuropathology of Sporadic Parkinson Disease before the Appearance of Parkinsonism: Preclinical Parkinson Disease. J. Neural Transm..

[6] Chu W., Chepetan A., Zhou D., Shoghi K.I., Xu J., Dugan L.L., Gropler R.J., Mintun M.A., Mach R.H. (2014). Development of a PET Radiotracer for Non-Invasive Imaging of the Reactive Oxygen Species, Superoxide, in Vivo. Org. Biomol. Chem..

[7] Hou C., Hsieh C.-J., Li S., Lee H., Graham T.J., Xu K., Weng C.-C., Doot R.K., Chu W., Chakraborty S.K. (2018). Development of a Positron Emission Tomography Radiotracer for Imaging Elevated Levels of Superoxide in Neuroinflammation. ACS Chem. Neurosci..

[8] Cuddihy S.L., Ali S.S., Musiek E.S., Lucero J., Kopp S.J., Morrow J.D., Dugan L.L. (2008). Prolonged Alpha-Tocopherol Deficiency Decreases Oxidative Stress and Unmasks Alpha-Tocopherol-Dependent Regulation of Mitochondrial Function in the Brain. J. Biol. Chem..

[9] Beckstead M.J., Howell R.D. (2021). Progressive Parkinsonism Due to Mitochondrial Impairment: Lessons from the MitoPark Mouse Model. Exp. Neurol..

[10] Liu H., Zhen C., Xie J., Luo Z., Zeng L., Zhao G., Lu S., Zhuang H., Fan H., Li X. (2024). TFAM Is an Autophagy Receptor That Limits Inflammation by Binding to Cytoplasmic Mitochondrial DNA. Nat. Cell Biol..

[11] West A.P., Khoury-Hanold W., Staron M., Tal M.C., Pineda C.M., Lang S.M., Bestwick M., Duguay B.A., Raimundo N., MacDuff D.A. (2015). Mitochondrial DNA Stress Primes the Antiviral Innate Immune Response. Nature.

[12] Langley M., Ghosh A., Charli A., Sarkar S., Ay M., Luo J., Zielonka J., Brenza T., Bennett B., Jin H. (2017). Mito-Apocynin Prevents Mitochondrial Dysfunction, Microglial Activation, Oxidative Damage, and Progressive Neurodegeneration in MitoPark Transgenic Mice. Antioxid. Redox Signal..

[13] Ekstrand M.I., Terzioglu M., Galter D., Zhu S., Hofstetter C., Lindqvist E., Thams S., Bergstrand A., Hansson F.S., Trifunovic A. (2007). Progressive Parkinsonism in Mice with Respiratory-Chain-Deficient Dopamine Neurons. Proc. Natl. Acad. Sci. USA.

[14] Wang C., Guan Y., Lv M., Zhang R., Guo Z., Wei X., Du X., Yang J., Li T., Wan Y. (2018). Manganese Increases the Sensitivity of the cGAS-STING Pathway for Double-Stranded DNA and Is Required for the Host Defense against DNA Viruses. Immunity.

[15] Sliter D.A., Martinez J., Hao L., Chen X., Sun N., Fischer T.D., Burman J.L., Li Y., Zhang Z., Narendra D.P. (2018). Parkin and PINK1 Mitigate STING-Induced Inflammation. Nature.

[16] González-Hunt C.P., Leung M.C.K., Bodhicharla R.K., McKeever M.G., Arrant A.E., Margillo K.M., Ryde I.T., Cyr D.D., Kosmaczewski S.G., Hammarlund M. (2014). Exposure to Mitochondrial Genotoxins and Dopaminergic Neurodegeneration in Caenorhabditis Elegans. PLoS ONE.

[17] Langley M.R., Ghaisas S., Ay M., Luo J., Palanisamy B.N., Jin H., Anantharam V., Kanthasamy A., Kanthasamy A.G. (2018). Manganese Exposure Exacerbates Progressive Motor Deficits and Neurodegeneration in the MitoPark Mouse Model of Parkinson’s Disease: Relevance to Gene and Environment Interactions in Metal Neurotoxicity. NeuroToxicology.

[18] Iliev A.I., Stringaris A.K., Nau R., Neumann H. (2004). Neuronal Injury Mediated via Stimulation of Microglial Toll-like Receptor-9 (TLR9). FASEB J..

[19] Sharpley M.S., Marciniak C., Eckel-Mahan K., McManus M., Crimi M., Waymire K., Lin C.S., Masubuchi S., Friend N., Koike M. (2012). Heteroplasmy of Mouse mtDNA Is Genetically Unstable and Results in Altered Behavior and Cognition. Cell.

[20] Morrow R.M., Picard M., Derbeneva O., Leipzig J., McManus M.J., Gouspillou G., Barbat-Artigas S., Dos Santos C., Hepple R.T., Murdock D.G. (2017). Mitochondrial Energy Deficiency Leads to Hyperproliferation of Skeletal Muscle Mitochondria and Enhanced Insulin Sensitivity. Proc. Natl. Acad. Sci. USA.

[21] Luong T.N., Carlisle H.J., Southwell A., Patterson P.H. (2011). Assessment of Motor Balance and Coordination in Mice Using the Balance Beam. JoVE.

[22] Ghaisas S., Langley M.R., Palanisamy B.N., Dutta S., Narayanaswamy K., Plummer P.J., Sarkar S., Ay M., Jin H., Anantharam V. (2019). MitoPark Transgenic Mouse Model Recapitulates the Gastrointestinal Dysfunction and Gut-Microbiome Changes of Parkinson’s Disease. NeuroToxicology.

[23] Mirrione M.M., Schiffer W.K., Fowler J.S., Alexoff D.L., Dewey S.L., Tsirka S.E. (2007). A Novel Approach for Imaging Brain-Behavior Relationships in Mice Reveals Unexpected Metabolic Patterns during Seizures in the Absence of Tissue Plasminogen Activator. Neuroimage.

[24] Sawiak S.J., Williams G.B., Morton A.J., Carpenter T.A. SPMMouse: A New Toolbox for SPM in the Animal Brain. Proceedings of the ISMRM 17th Scientific Meeting & Exhibition.

[25] Hsieh C.-J., Hou C., Zhu Y., Lee J.Y., Kohli N., Gallagher E., Xu K., Lee H., Li S., McManus M.J. (2022). [(18)F]ROStrace Detects Oxidative Stress in Vivo and Predicts Progression of Alzheimer’s Disease Pathology in APP/PS1 Mice. EJNMMI Res..

[26] Hosoi R., Sato S., Shukuri M., Fujii Y., Todoroki K., Arano Y., Sakai T., Inoue O. (2019). A Simple Ex Vivo Semiquantitative Fluorescent Imaging Utilizing Planar Laser Scanner: Detection of Reactive Oxygen Species Generation in Mouse Brain and Kidney. Mol. Imaging.

[27] Andrews Z.B., Horvath B., Barnstable C.J., Elseworth J., Yang L., Beal M.F., Roth R.H., Matthews R.T., Horvath T.L. (2005). Horvath Uncoupling Protein-2 Is Critical for Nigral Dopamine Cell Survival in a Mouse Model of Parkinson’s Disease. J. Neurosci..

[28] Cope E.C., Briones B.A., Brockett A.T., Martinez S., Vigneron P.-A., Opendak M., Wang S.S.-H., Gould E. (2016). Immature Neurons and Radial Glia, But Not Astrocytes or Microglia, Are Altered in Adult Cntnap2 and Shank3 Mice, Models of Autism. eNeuro.

[29] Guarnieri J.W., Lie T., Albrecht Y.E.S., Hewin P., Jurado K.A., Widjaja G.A., Zhu Y., McManus M.J., Kilbaugh T.J., Keith K. (2024). Mitochondrial Antioxidants Abate SARS-COV-2 Pathology in Mice. Proc. Natl. Acad. Sci. USA.

[30] Armstrong M.J., Okun M.S. (2020). Diagnosis and Treatment of Parkinson Disease: A Review. JAMA.

[31] Cerri S., Mus L., Blandini F. (2019). Parkinson’s Disease in Women and Men: What’s the Difference?. J. Park. Dis..

[32] Shulman L.M., Bhat V. (2006). Gender Disparities in Parkinson’s Disease. Expert. Rev. Neurother..

[33] Martínez de Toda I., González-Sánchez M., Díaz-Del Cerro E., Valera G., Carracedo J., Guerra-Pérez N. (2023). Sex Differences in Markers of Oxidation and Inflammation. Implications for Ageing. Mech. Ageing Dev..

[34] Antzoulatos E., Jakowec M.W., Petzinger G.M., Wood R.I. (2010). Sex Differences in Motor Behavior in the MPTP Mouse Model of Parkinson’s Disease. Pharmacol. Biochem. Behav..

[35] Bispo J.M.M., Melo J.E.C., Gois A.M., Leal P.C., Lins L.C.R.F., Souza M.F., Medeiros K.A.A.L., Ribeiro A.M., Silva R.H., Marchioro M. (2019). Sex Differences in the Progressive Model of Parkinsonism Induced by Reserpine in Rats. Behav. Brain Res..

[36] Giangrasso D.M., Furlong T.M., Keefe K.A. (2020). Characterization of Striatum-Mediated Behavior and Neurochemistry in the DJ-1 Knock-out Rat Model of Parkinson’s Disease. Neurobiol. Dis..

[37] Walrand S., Valeix S., Rodriguez C., Ligot P., Chassagne J., Vasson M.-P. (2003). Flow Cytometry Study of Polymorphonuclear Neutrophil Oxidative Burst: A Comparison of Three Fluorescent Probes. Clin. Chim. Acta.

[38] Gomes A., Fernandes E., Lima J.L.F.C. (2005). Fluorescence Probes Used for Detection of Reactive Oxygen Species. J. Biochem. Biophys. Methods.

[39] Dugan L.L., Ali S.S., Shekhtman G., Roberts A.J., Lucero J., Quick K.L., Behrens M.M. (2009). IL-6 Mediated Degeneration of Forebrain GABAergic Interneurons and Cognitive Impairment in Aged Mice through Activation of Neuronal NADPH Oxidase. PLoS ONE.

[40] Münzel T., Afanas’ev I.B., Kleschyov A.L., Harrison D.G. (2002). Detection of Superoxide in Vascular Tissue. Arterioscler. Thromb. Vasc. Biol..

[41] Tansey M.G., Goldberg M.S. (2010). Neuroinflammation in Parkinson’s Disease: Its Role in Neuronal Death and Implications for Therapeutic Intervention. Neurobiol. Dis..

[42] Mathys H., Adaikkan C., Gao F., Young J.Z., Manet E., Hemberg M., De Jager P.L., Ransohoff R.M., Regev A., Tsai L.-H. (2017). Temporal Tracking of Microglia Activation in Neurodegeneration at Single-Cell Resolution. Cell Rep..

[43] Vandenbark A.A., Offner H., Matejuk S., Matejuk A. (2021). Microglia and Astrocyte Involvement in Neurodegeneration and Brain Cancer. J. Neuroinflamm..

[44] Zotova E., Bharambe V., Cheaveau M., Morgan W., Holmes C., Harris S., Neal J.W., Love S., Nicoll J.A.R., Boche D. (2013). Inflammatory Components in Human Alzheimer’s Disease and after Active Amyloid-Β42 Immunization. Brain.

[45] Holness C.L., Simmons D.L. (1993). Molecular Cloning of CD68, a Human Macrophage Marker Related to Lysosomal Glycoproteins. Blood.

[46] Lin M.T., Beal M.F. (2006). Mitochondrial Dysfunction and Oxidative Stress in Neurodegenerative Diseases. Nature.

[47] Picard M., McManus M.J., Gray J.D., Nasca C., Moffat C., Kopinski P.K., Seifert E.L., McEwen B.S., Wallace D.C. (2015). Mitochondrial Functions Modulate Neuroendocrine, Metabolic, Inflammatory, and Transcriptional Responses to Acute Psychological Stress. Proc. Natl. Acad. Sci. USA.

[48] Butterfield D.A., Boyd-Kimball D. (2020). Mitochondrial Oxidative and Nitrosative Stress and Alzheimer Disease. Antioxidants.

[49] Dhapola R., Beura S.K., Sharma P., Singh S.K., HariKrishnaReddy D. (2024). Oxidative Stress in Alzheimer’s Disease: Current Knowledge of Signaling Pathways and Therapeutics. Mol. Biol. Rep..

[50] McManus M.J., Murphy M.P., Franklin J.L. (2011). The Mitochondria-Targeted Antioxidant MitoQ Prevents Loss of Spatial Memory Retention and Early Neuropathology in a Transgenic Mouse Model of Alzheimer’s Disease. J. Neurosci..

[51] Jiang Z., Wang W., Perry G., Zhu X., Wang X. (2015). Mitochondrial Dynamic Abnormalities in Amyotrophic Lateral Sclerosis. Transl. Neurodegener..

[52] Zhao J., Wang X., Huo Z., Chen Y., Liu J., Zhao Z., Meng F., Su Q., Bao W., Zhang L. (2022). The Impact of Mitochondrial Dysfunction in Amyotrophic Lateral Sclerosis. Cells.

[53] Huang L., Li Z., Zhang X. (2022). Radiotracers for Nuclear Imaging of Reactive Oxygen Species: Advances Made So Far. Bioconjug. Chem..

[54] Sivapackiam J., Liao F., Zhou D., Shoghi K.I., Gropler R.J., Gelman A.E., Sharma V. (2020). Galuminox: Preclinical Validation of a Novel PET Tracer for Non-Invasive Imaging of Oxidative Stress in Vivo. Redox Biol..

[55] Ikawa M., Okazawa H., Arakawa K., Kudo T., Kimura H., Fujibayashi Y., Kuriyama M., Yoneda M. (2009). PET Imaging of Redox and Energy States in Stroke-like Episodes of MELAS. Mitochondrion.

[56] Tada T., Mizuno Y., Shibata Y., Yasui H., Kuge Y. (2024). Application of Copper (I) Selective Ligands for PET Imaging of Reactive Oxygen Species through Metabolic Trapping. Nucl. Med. Biol..

[57] Gallagher E., Hou C., Zhu Y., Hsieh C.-J., Lee H., Li S., Xu K., Henderson P., Chroneos R., Sheldon M. (2024). Positron Emission Tomography with [(18)F]ROStrace Reveals Progressive Elevations in Oxidative Stress in a Mouse Model of Alpha-Synucleinopathy. Int. J. Mol. Sci..

[58] McManus M.J., Murphy M.P., Franklin J.L. (2014). Mitochondria-Derived Reactive Oxygen Species Mediate Caspase-Dependent and -Independent Neuronal Deaths. Mol. Cell. Neurosci..

[59] Cristóvão A.C., Guhathakurta S., Bok E., Je G., Yoo S.D., Choi D.-H., Kim Y.-S. (2012). NADPH Oxidase 1 Mediates α-Synucleinopathy in Parkinson’s Disease. J. Neurosci..

[60] Nilsen J., Irwin R.W., Gallaher T.K., Brinton R.D. (2007). Estradiol In Vivo Regulation of Brain Mitochondrial Proteome. J. Neurosci..

[61] Rettberg J.R., Yao J., Brinton R.D. (2014). Estrogen: A Master Regulator of Bioenergetic Systems in the Brain and Body. Front. Neuroendocrinol..

[62] Borrás C., Gambini J., López-Grueso R., Pallardó F.V., Viña J. (2010). Direct Antioxidant and Protective Effect of Estradiol on Isolated Mitochondria. Biochim. Biophys. Acta (BBA)-Mol. Basis Dis..

[63] Guillot-Sestier M.-V., Araiz A.R., Mela V., Gaban A.S., O’Neill E., Joshi L., Chouchani E.T., Mills E.L., Lynch M.A. (2021). Microglial Metabolism Is a Pivotal Factor in Sexual Dimorphism in Alzheimer’s Disease. Commun. Biol..

[64] Thakkar R., Wang R., Wang J., Vadlamudi R.K., Brann D.W. (2018). 17β-Estradiol Regulates Microglia Activation and Polarization in the Hippocampus Following Global Cerebral Ischemia. Oxidative Med. Cell. Longev..

[65] Beheshti I., Booth S., Ko J.H. (2024). Differences in Brain Aging between Sexes in Parkinson’s Disease. NPJ Park. Dis..

[66] De Miranda B.R., Fazzari M., Rocha E.M., Castro S., Greenamyre J.T. (2019). Sex Differences in Rotenone Sensitivity Reflect the Male-to-Female Ratio in Human Parkinson’s Disease Incidence. Toxicol. Sci..

[67] Misiak M., Beyer C., Arnold S. (2010). Gender-Specific Role of Mitochondria in the Vulnerability of 6-Hydroxydopamine-Treated Mesencephalic Neurons. Biochim. Biophys. Acta.

[68] Lee J., Pinares-Garcia P., Loke H., Ham S., Vilain E., Harley V.R. (2019). Sex-Specific Neuroprotection by Inhibition of the Y-Chromosome Gene, SRY, in Experimental Parkinson’s Disease. Proc. Natl. Acad. Sci. USA.

[69] Won J.-H., Park S., Hong S., Son S., Yu J.-W. (2015). Rotenone-Induced Impairment of Mitochondrial Electron Transport Chain Confers a Selective Priming Signal for NLRP3 Inflammasome Activation. J. Biol. Chem..

[70] Chen L., Na R., Boldt E., Ran Q. (2015). NLRP3 Inflammasome Activation by Mitochondrial Reactive Oxygen Species Plays a Key Role in Long-Term Cognitive Impairment Induced by Paraquat Exposure. Neurobiol. Aging.

[71] Gao X., Hu X., Qian L., Yang S., Zhang W., Zhang D., Wu X., Fraser A., Wilson B., Flood P.M. (2008). Formyl-Methionyl-Leucyl-Phenylalanine–Induced Dopaminergic Neurotoxicity via Microglial Activation: A Mediator between Peripheral Infection and Neurodegeneration?. Environ. Health Perspect..

[72] Miller Y.I., Choi S.-H., Wiesner P., Fang L., Harkewicz R., Hartvigsen K., Boullier A., Gonen A., Diehl C.J., Que X. (2011). Oxidation-Specific Epitopes Are Danger-Associated Molecular Patterns Recognized by Pattern Recognition Receptors of Innate Immunity. Circ. Res..

[73] Picard M., McManus M.J., Reeve A.K., Simcox E.M., Duchen M.R., Turnbull D.M. (2016). Mitochondrial Signaling and Neurodegeneration. Mitochondrial Dysfunction in Neurodegenerative Disorders.

[74] Woo D.K., Green P.D., Santos J.H., D’Souza A.D., Walther Z., Martin W.D., Christian B.E., Chandel N.S., Shadel G.S. (2012). Mitochondrial Genome Instability and ROS Enhance Intestinal Tumorigenesis in APC(Min/+) Mice. Am. J. Pathol..

[75] Shimada K., Crother T.R., Karlin J., Dagvadorj J., Chiba N., Chen S., Ramanujan V.K., Wolf A.J., Vergnes L., Ojcius D.M. (2012). Oxidized Mitochondrial DNA Activates the NLRP3 Inflammasome during Apoptosis. Immunity.

[76] Gordon R., Albornoz E.A., Christie D.C., Langley M.R., Kumar V., Mantovani S., Robertson A.A.B., Butler M.S., Rowe D.B., O’Neill L.A. (2018). Inflammasome Inhibition Prevents α-Synuclein Pathology and Dopaminergic Neurodegeneration in Mice. Sci. Transl. Med..

