# Supplementary material for: PET Imaging with [18F]ROStrace Detects Oxidative Stress and Predicts Parkinson’s Disease Progression in Mice
Source: Antioxidants (Basel). 2024 Oct 12;13(10):1226. doi: 10.3390/antiox13101226 (PMC11504722; doi:10.3390/antiox13101226)
Supplement: Supplementary file 1 [file antioxidants-13-01226-s001.zip › Supplementary Materials.pdf]

Supplemental Figures

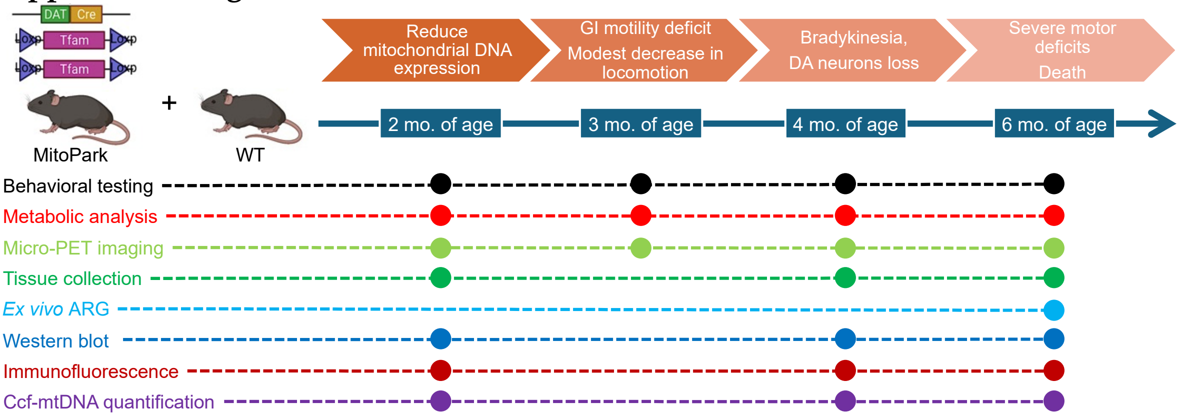

Figure S1. Schematic illustration of experimental scheme

MitoPark animals are generated by selective deletion of the transcription factor A mitochondrial (Tfam) from dopaminergic (DA) neurons using the DAT-CRE system. The natural history of PD-like disease in MitoPark mice is shown from 2 – 6 mo. of age. Metabolic, neurobehavioral, and PET imaging analysis was performed throughout the course of PD-like disease in MitoPark animals and age-matched controls. Blood and brain were collected at 2, 4, and 6 mo. of age. Western blots and immunofluorescence were conducted on brain tissue and circulating cell free-mitochondrial DNA (ccf-mtDNA) was quantified from plasma at each of these time points. DAT: Dopamine Transporter.

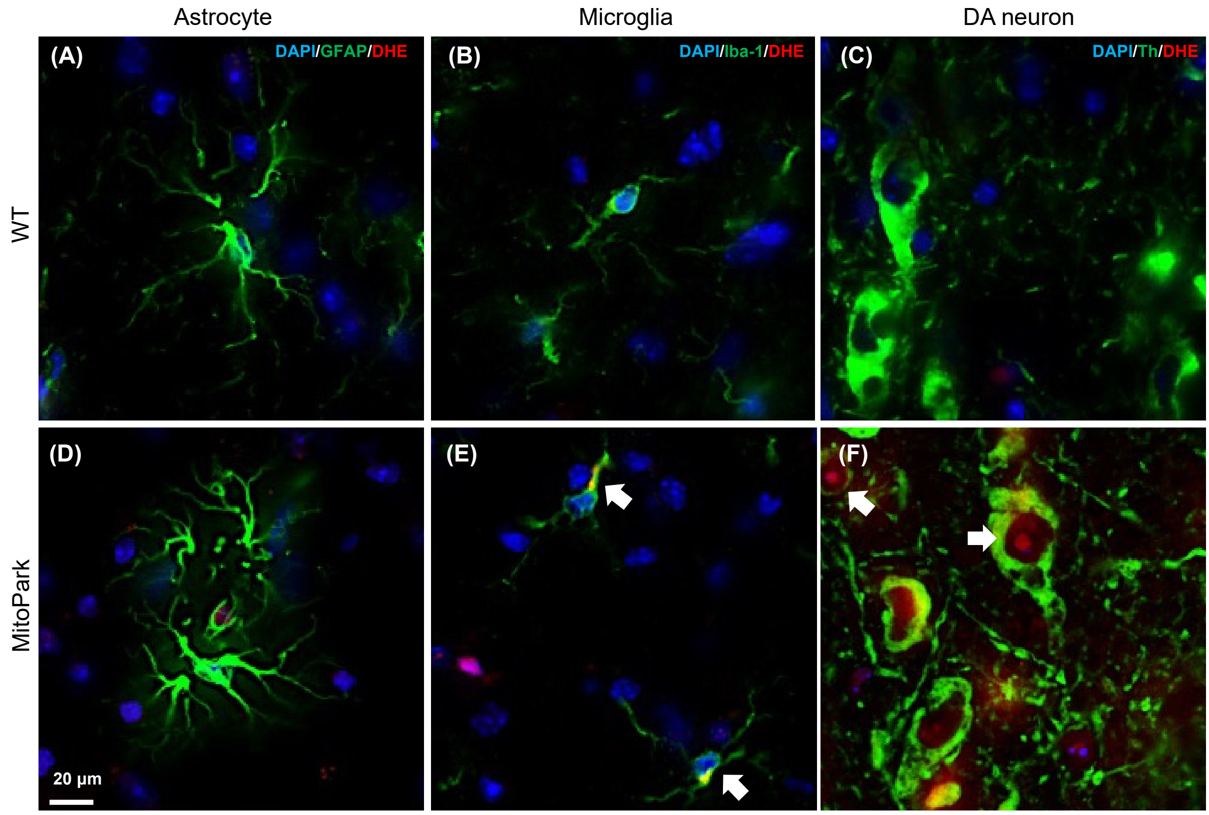

Figure S2. Colocalization of oxidized DHE in the MitoPark microglia and DA neurons.

Colocalization of oxidized DHE (red) with major brain cell types (green) in the SN region of 4 mo. WT (A, B, C) and MitoPark (D, E, F). White arrows indicate oxidized DHE colocalized with MitoPark microglia (E) and DA neurons (F) in the SN. Scale bar = 20 µm.

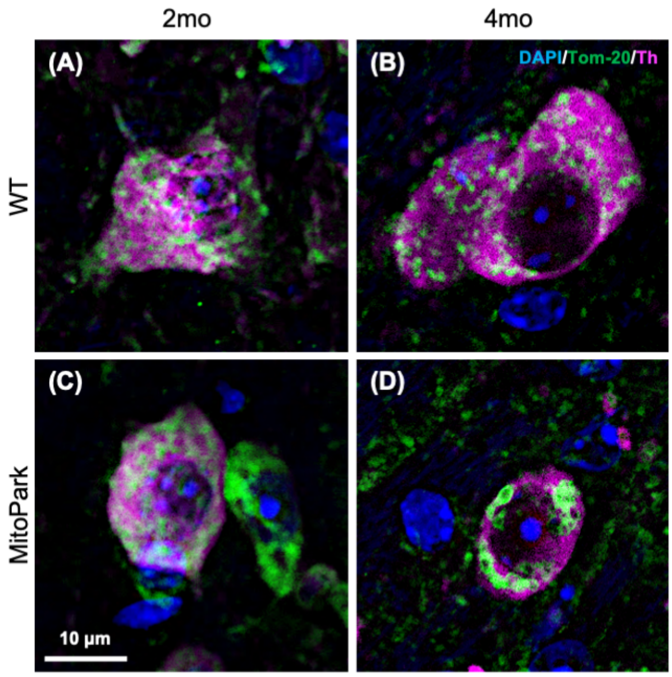

Figure S3. Abnormal mitochondria in TH+ neurons of young MitoPark mice.
(**A-D**) Representative images showing an abnormal mitochondrial network in the TH+ neurons in the MitoPark SN (green; **C**., **D**.) compared to WT (**A**., **B**.).

Table S1. Information for primary antibodies

Name	Species	Cat number	Supplier	Application
Anti-Glial Fibrillary Acidic Protein (GFAP)	Rabbit	AB5804	Millipore Sigma	IF: 1:200 in 1% BSA in PBS
IBA-1	Goat	AB5076	Abcam	IF: 1:200 in 1% BSA in PBS
IBA-1	Rabbit	019-19741	Wako	IF: 1:200 in 1% BSA in PBS
CD68	Rabbit	97778S	Cell Signaling	IF: 1:200 in 1% BSA in PBS
Tyrosine Hydroxylase (TH)	Mouse	MAB318	EMD Millipore	IF: 1:200 in 1% BSA in PBS
Tyrosine Hydroxylase (TH)	Rabbit	PA5-85167	ThermoFisher Scientific	IF: 1:200 in 1% BSA in PBS WB: 1:1000 1% BSA in PBS
Tom-20	Mouse	NC0563977	Fisher Scientific	IF: 1:200 in 1% BSA in PBS
GAPDH	Mouse	MA5-15738	ThermoFisher Scientific	WB: 1:2000 3% BSA in PBS
Alexa Fluor 488	Rabbit	A11008	ThermoFisher Scientific	IF: 1:300 in 1% BSA in PBS
Alexa Fluor 647	Mouse	A21235	ThermoFisher Scientific	IF: 1:300 in 1% BSA in PBS
Alexa Flour 594	Donkey	A-11058	ThermoFisher Scientific	IF: 1:300 in 1% BSA in PBS
IRDye 800CW	Donkey	926-32212 926-32213	LI-COR	WB: 1:20,000 3% BSA in PBS
IRDye 680RD	Donkey	926-68072 926-68073	LI-COR	WB: 1:10,000 3% BSA in PBS

Abbreviations

Aβ amyloid-beta
AD Alzheimer's disease
ALS Amyotrophic lateral sclerosis
ARG autoradiography
ATP adenosine triphosphate
BBB Blood Brain Barrier
Ccf-mtDNA Circulating cell-free mitochondrial DNA
CLAMS Comprehensive Lab Animal Monitoring System
CNS Central Nervous System
CT Computed Tomography scan
DA Dopaminergic
DAMPs Damage-associated molecular patterns
DNA Deoxyribonucleic acid
DHE Dihydroethidium
ETC Electron transport chain
FTLD Frontotemporal lobar dementia
GFAP Glial Fibrillary Acidic Protein
iNOS induced Nitric Oxide Synthase
IR Infrared Beam
MP MitoPark
MRI Magnetic resonance imaging
mtDNA mitochondrial DNA
ND Neurodegenerative diseases
NOS Nitric Oxide Synthase
NOX NADPH oxidase
PD Parkinson's disease
PET Positron emission tomography
RER Respiratory exchange ratio
RNA Ribonucleic acid
ROS Reactive Oxygen Species
SN Substantia Nigra
SPM Statistical parametric mapping
SUV Standardized uptake values
Tfam Transcription factor A mitochondrial
TH Tyrosine hydroxylase
VM Ventral Midbrain
VOI Volume of Interest
WB Western Blot
WT Wild Type
